# Three-dimensional postoperative accuracy of extra-articular forearm osteotomies using CT-scan based patient-specific surgical guides

**DOI:** 10.1186/s12891-015-0793-x

**Published:** 2015-11-04

**Authors:** Lazaros Vlachopoulos, Andreas Schweizer, Matthias Graf, Ladislav Nagy, Philipp Fürnstahl

**Affiliations:** Computer Assisted Research and Development Group, Balgrist University Hospital, University of Zurich, Zurich, Switzerland; Department of Orthopaedics, Balgrist University Hospital, University of Zurich, Zurich, Switzerland

**Keywords:** Computer-assisted planning, Patient-specific, Drill guides, Cutting guides, Forearm, Osteotomies, Additive manufacturing, Angular-stable locking plate

## Abstract

**Background:**

Computer assisted corrective osteotomy of the diaphyseal forearm and the distal radius based on computer simulation and patient-specific guides has been described as a promising technique for accurate reconstruction of forearm deformities. Thereby, the intraoperative use of patient-specific drill and cutting guides facilitate the transfer of the preoperative plan to the surgery. However, the difference between planned and performed reduction is difficult to assess with conventional radiographs. The aim of this study was to evaluate the accuracy of this surgical technique based on postoperative three-dimensional (3D) computed tomography (CT) data.

**Methods:**

Fourteen patients (mean age 23.2 (range, 12-58) years) with an extra-articular deformity of the forearm had undergone computer assisted corrective osteotomy with the healthy anatomy of the contralateral uninjured side as a reconstruction template. 3D bone surface models of the pathological and contralateral side were created from CT data for the computer simulation. Patient-specific drill and cutting guides including the preoperative planned screw direction of the angular-stable locking plates and the osteotomy planes were used for the intraoperative realization of the preoperative plan. There were seven opening wedge osteotomies and nine closing wedge (or single-cut) osteotomies performed.

Eight-ten weeks postoperatively CT scans were obtained to assess bony consolidation and additionally used to generate a 3D model of the forearm. The simulated osteotomies- preoperative bone models with simulated correction - and the performed osteotomies - postoperative bone models – were analyzed for residual differences in 3D alignment.

**Results:**

On average, a significant higher residual rotational deformity was observed in opening wedge osteotomies (8.30° ± 5.35°) compared to closing wedge osteotomies (3.47° ± 1.09°). The average residual translation was comparable small in both groups, i.e., below 1.5 mm and 1.1 mm for opening and closing wedge osteotomies, respectively.

**Conclusions:**

The technique demonstrated high accuracy in performing closing wedge (or single-cut) osteotomies. However, for opening wedge osteotomies with extensive lengthening, probably due to the fact that precise reduction was difficult to achieve or maintain, the final corrections were less accurate.

## Background

Posttraumatic deformities of the forearm bones (radius shaft, ulna shaft or distal radius) may cause impairment of the forearm function, i.e. limited range of motion (ROM), especially decreased pro- and supination and a painful or instable distal radioulnar joint [1, 2]. Corrective osteotomies with anatomical reduction and restoration of the normal anatomy are regularly considered in symptomatic cases in order to relieve pain and/or to improve the ROM. Dependent on the deformity, a bone wedge has to be either removed (i.e., closing wedge osteotomy) or a gap has to be created (i.e., opening wedge osteotomy) to achieve the desired reduction [3].

Computer assisted corrective osteotomy of the diaphyseal forearm and the distal radius based on computer simulation has been described as a promising technique for accurate reconstruction of forearm deformities. Three-dimensional (3D) bone models are therefore frequently extracted from computed tomography (CT) scans [4–11]. The planned correction is simulated by manipulating the bone models in virtual 3D space. However, the transfer of the final planned correction into the intraoperative realization remains technically challenging. With the increasing use of patient- specific guides several methods have been described suitable for this purpose. Croitoru et al. [12] first introduced the idea of integrating a digitized model of an osteosynthesis plate into the preoperative plan [12] in order to predrill the screw holes intraoperatively according to the preoperative plan previous to the osteotomy. After osteotomy, by inserting the screw through the plate into the predrilled holes an indirect – automatic - reduction can be achieved. More recently, Miyake et al. [13] and Kunz et al. [8] further developed this technique by using patient-specific drill and cutting guides in order to avoid a navigation system used by Croitoru et al. [12].

As exact restoration of the normal anatomy is essential for a good clinical outcome [14], the reproducibility and accuracy of the technique needs to be assessed. However, most studies have relied on 2D based postoperative evaluation, disregarding the 3D characteristic of the residual deformities.

The purpose of the study was to assess the 3D accuracy of the performed corrective osteotomies of the forearm with patient-specific guides and integrated digitized model of an angular- stable locking plate into the preoperative plan compared to the planned correction.

## Methods

Between January and September 2013, a total of 14 patients (mean age 23.2 (range 12-58) years, 8 female) with malunited fractures of the radius or ulna were treated by corrective osteotomy at our institution. Demographic data are summarized in Table 1. Inclusion criteria were patients with a symptomatic extra-articular malunion, according to the criteria of Leong et al. [9] and McQueen et al. [15], with restriction in flexion and pro-/supination, radial shortening due to the malunion or premature close of the epiphyseal growth plates as well as pain due to distal radioulnar instability more than three months after initial trauma. Exclusion criteria were an intra-articular malunion and pathology of the contralateral forearm. Informed consent was obtained from all patients preoperatively (for patients under 18 years in addition parental consent was obtained) regarding the permission to report on their medical history, demographics, characteristics and postoperative results and to publish personal information (as contained in Table 1). Ethical approval was obtained from the ethical committee of the Canton of Zurich for retrospective data analysis. The patients were assigned to one of two groups according to the type of osteotomy. Group I represented corrective osteotomies with the bone fragments being in direct contact at the osteotomy site after reduction, i.e., closing wedge and single-cut [16] osteotomies. Contrary, opening wedge osteotomies were assigned to Group II. All patients with an osteotomy of the radius shaft or ulna shaft were assigned to Group I, since the surgeons avoid due to concerns about the healing of the osteotomies an open wedge in this region. All patients except one with an osteotomy of the distal radius were assigned to Group II, since most of the patients with a distal radius malunion had a various degree of radial shortening. Osteotomies were preoperatively planned in 3D and patient-specific drill and cutting guides were applied intraoperatively to perform the correction.

**Table 1:** Demographic and clinical data are given for each patient

Group	Patient	Age (years)	Gender	Side	Injury to surgery (months)	Osteotomy	Occupation
Group I	1	58	female	left	35	closing wedge distal radius	saleswoman
						single cut ulna	
	2	14	male	left	20	closing wedge radius shaft	student
						closing wedge ulna shaft	
	3	18	female	right	53	closing wedge radius shaft	saleswoman
	4	18	female	right	61	closing wedge radius shaft	logistician
	5	21	male	right	6	closing wedge ulna shaft	merchant
	6	28	female	left	91	single cut ulna	dental assistent
	7	23	male	right	90	single cut ulna	mechanic
Group II	8	35	male	right	9	open wedge distal radius	heating engineer
	9	15	female	left	33	open wedge distal radius	student
	10	12	female	left	9	open wedge distal radius	student
	11	14	female	right	40	open wedge distal radius	student
	12	13	female	left	no trauma date	open wedge distal radius	student
	13	15	male	left	5	open wedge distal radius	student
	14	41	male	right	13	open wedge distal radius	clerk

### Preoperative planning and surgical technique

**Fig. 1:**
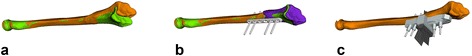
Preoperative Plan. Outline of the investigated computer-assisted planning approach. **a** Quantification of the malunion by superimposing the proximal part of the pathological bone (orange) with the mirrored contralateral bone (green). **b** Simulated reduction of the distal fragment (violet) and positioning of the fixation plate. The (beige) cylinders represent the angular-stable locking screws. **c** The screw models are transformed back to the pathological bone by applying the inverse transformation. The patient-specific drill and cutting guide (beige) is designed based on this information

3D triangular surface models of the radius and ulna of the pathological and contralateral uninjured side were generated by segmenting the bones from CT data (slice thickness 1 mm; 120 kV; Philips Brilliance 40 CT, Philips Healthcare, The Netherlands) using commercial software (Mimics, Materialise, Belgium). The CT protocol for corrective osteotomies of the forearm at our institution includes a contiguously scan of the whole forearm from the elbow to the radiocarpal joint. For segmentation, manual thresholding and region growing was applied. A senior surgeon performed the computer planning on a standard personal computer using the custom-made software application CASPA (Balgrist CARD AG, Zurich, Switzerland). To quantify the malunion in 3D, the model of the contralateral bone was mirrored and aligned with the pathological bone using the Iterative Closest Point (ICP) surface registration algorithm as previously described [5, 6] (Fig. 1a). After simulated osteotomy, the distal part was reduced to the contralateral bone using ICP. Next, a 3D model of the angular-stable locking plate was positioned on the bone surface. Dependent on the malunion, one of the following implants was chosen by the surgeon: A 2.7 mm LCP plate (Synthes, Solothurn, Switzerland), a 2.7 mm LCP ulnar shortening osteotomy plate (Synthes), a 2.4 mm LCP distal radius plate (Synthes), or an Elegantus distal radius plate (Intercus Schweiz GmbH, Aarau, Switzerland). The plate models included cylinders representing the exact position and direction of the angular-stable locking screws (Fig. 1b) in relation to the plate. The screw models placed in the distal fragment were transformed with the previously registered distal part of the bone back to the pathological bone position by applying the inverse transformation. Thereby, during surgery the final direction of the screws can be predrilled previous to the osteotomy. Additionally, when a closing wedge osteotomy was planned, we used a metallic inlay that contains a cutting slit to guide the 0.4mm thick saw blade. The inlay was inserted into a dedicated frame in the guide body for alignment according to the planned osteotomy planes. In case of an open wedge osteotomy, since all osteotomies were complete with a resulting distraction gap, the exact position of the plane is not as important as for closing wedge or single cut osteotomies. Lastly, a surgical guide with drill sleeves and the dedicated frames for the metallic inlay was designed (Fig. 1c). To achieve a unique fit, the shape of the guide body was designed to contain irregular convex and concave parts covering the bone from different directions and the undersurface of the guide to be placed on the bone as an exact replication of the surface of the bone model. The guides were manufactured by Medacta International S.A. (Castel San Pietro, Switzerland) with a selective laser sintering device.

During surgery, periosteum and other soft tissues were striped from the bone to permit a unique fit of the guide. After placing the guide on its intended position, it was fixed with two K-wires. Next, the screw holes were predrilled and the osteotomy was performed, resulting in a proximal and distal fragment. The osteosynthesis plate was first fixed to the distal fragment using the predrilled holes and thereafter the proximal fragment was indirectly reduced by plate fixation. On both sides of the osteotomy at least three angular stable locking screws were placed. In case of a distal radial osteotomy in the distal fragment at least four angular stable locking screws were placed. In four cases (two in each group) the plate was preoperatively prebend based on the printout of a bone in the planned correction. Autogenous iliac cancellous bone or resected callous formation was used to fill the osteotomy gap in case of opening wedge osteotomies. Postoperative the patients were placed in a volar splint made of plaster for 6 weeks. Active assisted ROM exercises were initiated from the first postoperative day.

### Postoperative evaluation method

**Fig. 2:**
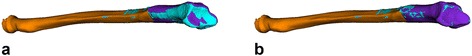
Postoperative Evaluation. The postoperative 3D evaluation is performed by comparing the preoperatively planned reduction (orange and violet fragments) with the bone model extracted from postoperative CT (cyan). **a** The proximal parts are superimposed. **b** The residual deformity is assessed by measuring the difference between the distal parts

**Fig. 3:**
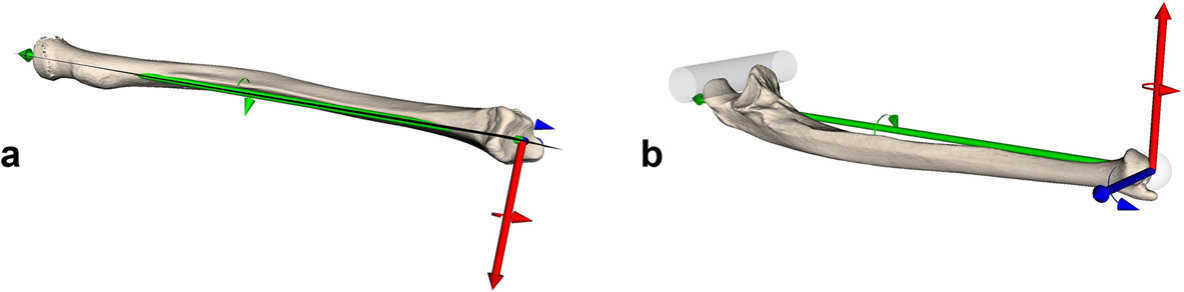
Definition of the anatomical coordinate system for radius (**a**) and ulna (**b**). Rotation around the x-axis (red) corresponds to a correction in the frontal (ulnarduction/radialduction) plane, around the y-axis (green) to a correction in the transverse plane (pronation/supination) and around the z-axis (blue) to a correction in sagittal (flexion/extension) plane. The coordinate system was adapted in that way that a positive rotation around the defined axis defined for both sides of the radius and ulna an ulnarduction, pronation and flexion, respectively

During follow up plain radiographs were regularly acquired at 4 weeks, 8–10 weeks, 4 months and 12 months. Additionally, CT scans were performed 8–10 weeks postoperatively with the same scanning protocol as preoperatively to assess bony consolidation. Consolidation was defined as disappearance of the visibility of the osteotomy lines on plain radiographs, continuous bone trabeculae at least half of the diameter of the bone on CT and absence of pain or swelling at the level of the osteotomy. The CT data were used to generate a 3D model of the postoperative bone, the implant, and the screws by applying the same segmentation method as in the preoperative planning. The bone parts proximal to the osteotomy were used as a common reference for comparing the postoperative bone with the preoperatively planned reduction. The proximal parts were registered using ICP as shown in Fig. 2a. Thereafter, the difference between planned and performed reduction (i.e., residual deformity) was quantified in all six degrees of freedom by computing the difference between the distal bone parts using ICP (Fig. 2b). The resulting 4×4 transformation matrix was decomposed in a rotational and translation part: The residual rotation was expressed in axis-angle representation and additionally as three constitutive rotations (i.e., Euler rotations) [17] around a standardized coordinate system as depicted in Fig. 3. Rotation around the x-, y-, and z-axis of the coordinate systems correspond to rotations in the frontal (ulnar-/radialduction), transverse plane (pronation/supination), and sagittal (flexion/extension) plane, respectively. The residual translation was expressed as a 3D vector describing the displacement with respect to the same coordinate axes.

### Statistical analysis

Group differences in baseline characteristics were compared using Mann–Whitney *U* test (for age) and unpaired *t* test (for the planned 3D correction angle). The residual rotational error and the clinical parameters (ROM and grip strength) were analyzed with an ANCOVA with the preoperative measurement as a covariable in the model. For graphical visualization Tukey boxplots were depicted with whiskers with maximum 1.5 interquartile range (ICR). The significance level was set at P < 0.05. The software R (Version 3.0; R Foundation, Vienna, Austria) was used for statistical evaluation.

## Results

Table 1 summarizes the demographic data of the patients for group I (i.e., closing wedge and single-cut osteotomies) and group II (i.e., opening wedge osteotomies). The median age of the patients in group I was 21.5 years (range 14–58 years), the median age of the patients in group II was 15 years (range 12–41 years). The median time from injury to surgery was 38 months (range 5–91 months). There was no significant difference in the baseline characteristics of both groups for age (Table 1; *P* = 0.22) and the amount of planned correction (Table 2; 19.94° ± 7.21° vs. 23.25° ± 10.48°, *P* = 0.49).

**Table 2:** Rotation is described by the 3D angle (axis-angle representation) and by Euler rotations around the anatomical coordinate system. Translation is given with respect to the anatomical coordinate system. Mean and standard deviation (SD) are based on absolute values

		Planned Correction							Residual error						
			Rotation (°)			Translation (mm)				Rotation (°)			Translation (mm)		
	Patient	3D angle	Flexion/Extension	Ulnar/Radial	Pronation/Supination	Palmar/Dorsal	Ulnar/Radial	Distal/Proximal	3D angle	Flexion/Extension	Ulnar/Radial	Pronation/Supination	Palmar/Dorsal	Ulnar/Radial	Distal/Proximal
**Group I**	1	**12.42**	11.99	1.24	−3.14	−0.15	−0.09	−2.17	**4.46**	2.07	3.53	−1.82	−0.80	−0.27	0.99
		**29.57**	−4.89	−4.63	28.61	0.75	0.95	0.69	**4.56**	−2.50	1.83	3.38	−1.32	−0.99	0.61
	2	**25.51**	20.51	15.00	1.18	−2.81	−2.03	−8.43	**4.92**	−1.50	1.67	4.40	1.42	0.04	−5.10
		**14.24**	−9.28	5.75	−8.70	0.38	−3.03	−1.43	**3.88**	−0.29	0.46	−3.84	−0.39	−2.17	−4.79
	3	**18.04**	14.16	−0.16	11.22	−0.51	−0.47	−1.59	**1.49**	−0.64	−1.34	−0.14	−0.30	0.14	0.10
	4	**12.15**	11.72	3.19	−0.60	−0.20	−0.14	−1.21	**3.16**	−2.56	0.12	1.85	0.28	1.06	0.37
	5	**13.71**	−10.06	9.35	1.04	−0.45	0.37	−1.15	**3.14**	−0.49	1.92	2.44	0.67	0.19	0.47
	6	**25.61**	0.86	−3.21	−25.37	0.15	0.18	−0.22	**2.72**	−1.29	0.98	2.20	0.10	−0.44	−0.50
	7	**28.20**	0.27	−1.07	28.18	−0.09	0.10	0.01	**2.87**	1.27	2.15	−1.43	−0.09	−0.79	0.38
	**Mean**	**19.94**	**9.31**	**4.85**	**12.00**	**0.61**	**0.82**	**1.88**	**3.47**	**1.40**	**1.56**	**2.39**	**0.60**	**0.68**	**1.48**
	**SD**	**7.21**	**6.47**	**4.73**	**12.11**	**0.85**	**1.04**	**2.55**	**1.09**	**0.84**	**1.01**	**1.31**	**0.50**	**0.68**	**1.98**
**Group II**	8	**22.79**	19.33	10.98	−7.39	−1.66	1.16	5.31	**7.94**	4.90	2.33	−5.90	0.10	1.79	0.86
	9	**10.49**	5.78	8.76	−0.71	0.55	0.36	7.66	**4.76**	3.63	0.56	−3.05	−1.99	−0.31	1.60
	10	**28.26**	24.34	−9.81	12.96	−0.54	0.81	9.08	**17.51**	13.08	−3.74	11.50	−0.92	−1.66	1.44
	11	**35.39**	7.42	32.61	9.84	0.09	1.15	17.93	**12.47**	4.06	10.86	4.25	−1.56	−1.21	2.17
	12	**23.58**	−10.46	−19.88	5.70	0.09	0.72	5.81	**9.38**	−2.24	−9.11	−0.21	1.70	1.14	−0.44
	13	**33.65**	−12.59	−16.12	−28.73	1.13	1.50	1.93	**3.36**	−0.36	−0.55	−3.29	−0.90	−1.08	0.08
	14	**8.57**	−2.51	0.48	8.19	4.43	4.57	1.83	**2.70**	1.14	2.44	−0.18	0.14	−0.35	0.53
	**Mean**	**23.25**	**10.34**	**12.46**	**12.71**	**1.07**	**1.30**	**6.20**	**8.30**	**3.84**	**3.97**	**3.73**	**0.94**	**1.04**	**0.94**
	**SD**	**10.48**	**7.73**	**10.18**	**8.87**	**1.53**	**1.42**	**5.49**	**5.35**	**4.23**	**4.12**	**3.88**	**0.75**	**0.57**	**0.74**

The bold values are based on absolute values

**Fig. 4:**
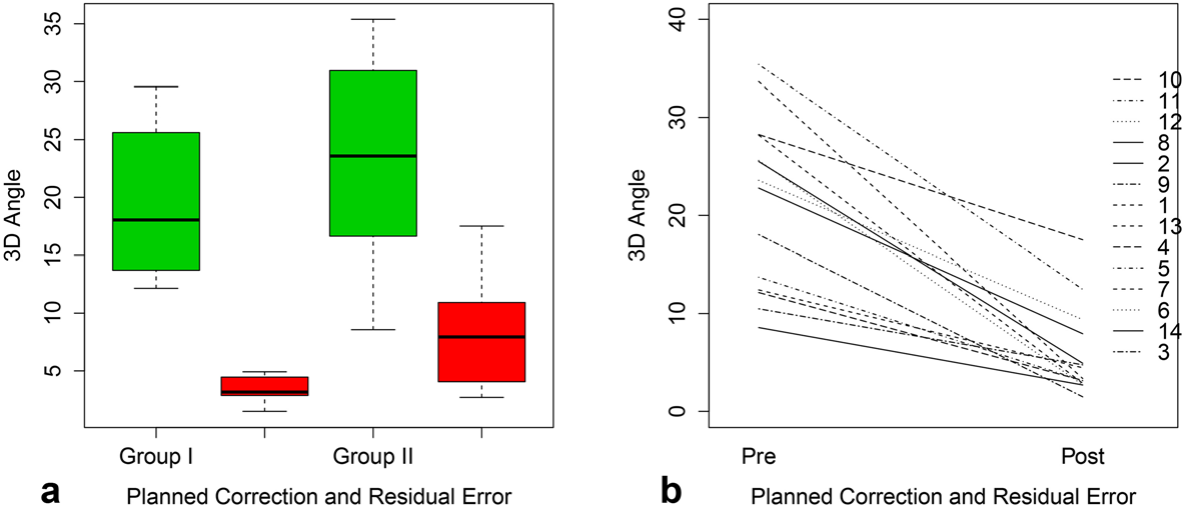
Planned Correction and Residual Error. **a** Boxplot illustrating the planned correction and residual deformity between group I and group II, **b** Each osteotomy is represented by a line. One endpoint is the planned correction, the other one is the residual deformity after surgery

**Fig. 5:**
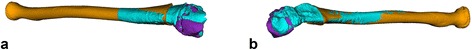
Postoperative 3D evaluation of the cases with highest residual error. Preoperatively planned reduction (orange and violet fragments) and postoperative result (cyan) of (**a**) case 10 and (**b**) case 11

Results of the preoperative planning and the postoperative accuracy evaluation are given in Table 2 and Fig. 4. On average, a significant higher (i.e., *P* = 0.03) difference in rotation between planned correction and postoperative result was observed in group II (8.30° ± 5.35°) compared to group I (3.47° ± 1.09°). The highest residual deformity was observed in case 10 and case 11 (Fig. 5). In these cases, also a deviation from the planned screw direction was located. For group I, the mean residual rotational deformity in sagittal, frontal, and transverse plane was 1.4° ± 0.84°, 1.56° ± 1.01°, and 2.39° ± 1.31°, respectively. For Group II, a residual rotation of 3.84° ± 4.23° in the sagittal plane, 3.97° ± 4.12° in the frontal plane, and 3.73° ± 3.88° in the transverse plane was measured. The average displacement between planned and performed reduction was between 0.5–1.48 mm and 0.94–1.04 mm for group I and group II, respectively.

Table 3 reveals the mean ROM and grip strength preoperative and change at the latest follow up. There was a significant difference between the preoperative and the postoperative values in flexion, pronation and in grip strength. The difference between the groups was not significant.

**Table 3:** Reveals the preoperative clinical values and the change at the latest follow up. P-values Change are given for the difference between preoperative and postoperative values, P-Values Group for the differences between the two groups

	Preoperative value	Chance	Chance	Group
	mean (± SD)	mean (± SD)	*P*-value *	*P*-value **
Flexion (°)	69 (±16)	7 (±11)	0.005 *	0.21
Extension (°)	75 (±14)	−3 (±14)	0.12	0.55
Pronation (°)	62 (±22)	10 (±19)	0.04 *	0.25
Supination (°)	65 (±26)	14 (±25)	0.33	0.90
Jamar (kg)	24 (±9)	5 (±6)	0.005 *	0.58

* P < 0.05 significant change of the preoperative ROM or grip strength

** P < 0.05 significant difference between the groups

All osteotomies healed after a mean time of 3.6 months (±1.8 months). The closing wedge osteotomies of group I healed after 4.4 months (±3.1 months) and the opening wedge osteotomies of group II after 3.4 (±2.0 months), respectively.

## Discussion

Accuracy in the preoperative quantification of a deformity and, subsequently, in the surgical reconstruction are crucial, because over- and under-correction is associated with loss of ROM and grip strength [14]. Therefore, computerized 3D comparison to the contralateral bone has been increasingly used to quantify complex malunions and the preoperative planning of corrective osteotomies. Different techniques, such as navigation systems [4, 12] or patient-specific guides were proposed to support the surgeon in performing the reduction exactly as preoperatively planned. Particularly, patient-specific guides are becoming popular, because they are easier to handle and more precise in vitro compared to navigation systems [18, 19]. Laboratory studies have reported average residual errors less than 1° and 1 mm for simulated osteotomies that used patient-specific guides [18, 19]. In these studies, the accuracy of the reduction was similar regardless whether K-wire based patient-specific reduction guides [19] or patient-specific drill guides based on the implant screw directions [18] were used.

Studies have already described promising clinical results of using the herein investigated technique [8, 12, 13, 20, 21]. Kunz et al. [8] reported on the radiological results of 8 patients treated by 3D planned distal radius opening wedge osteotomy using volar plating and patient-specific drill guides. The average volar tilt deviation after correction was 1.9° (±1.5°) and the average radial inclination deviation 1.8° (±0.8°). However, their evaluation was only based on intraoperative plane images using image intensifier. Moreover, the amount of the correction and the demographic data of the patients were not mentioned. Miyake et al. [13] analyzed the surgical outcome of open wedge osteotomy in ten patients using the same technique as in the current study with volar plates and autogenous iliac cancellous bone graft. The average difference to the contralateral unaffected side was 5.1° in volar tilt and 3.4° in radial inclination. Although Miyake et al. stated that 3D based methods may be more effective for complex deformities [22], the postoperative evaluation relied on 2D radiographs. The differences between 2D deformity assessment based on standard radiographs and 3D based methods was analyzed by Vroemen et al. [23]. Their study retrospectively investigated the residual deformity after conventionally performed corrective osteotomies. Only the 3D deformity measurements showed signification correlation with the clinical outcome.

**Fig. 6:**
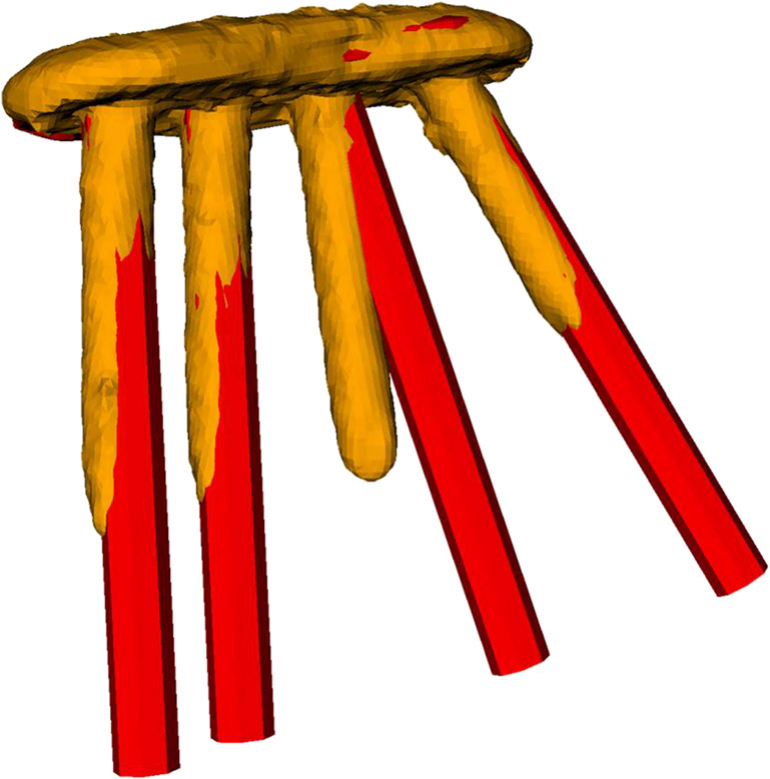
Locking plate with screws. Comparison of the planned screw direction (red) with the screws (orange) obtained from the postoperative CT with the highest residual error (case 10)

The current study reported on the accuracy of deformity correction using patient-specific drill guides by retrospectively analyzing available postoperative 3D CT data. In the closing wedge group, the residual rotational deformity observed in the three anatomical planes was between 1.4° to 2.4° on average. These results are comparable with in-vitro findings [18, 19]. Contrary, in group II residual deformities above 10° were measured in two cases. In these cases an excessive lengthening of the bone additionally to the rotational component was required (case 10: 17 mm; case 11: 10 mm) to achieve the planned reduction, resulting in very high soft tissue tensioning. Although a surgical spreader was used to temporary stabilize the fragment, it was difficult to perform the reduction solely based on the pre-drilled screw holes. The stress between the fragments might additionally cause the screws to lock into the plate slightly differently from the planned direction (see Fig. 6). Nevertheless, all residual deformities are considerable smaller compared to corrective osteotomies performed without patient-specific drill guides [23].

All patients with an osteotomy of the radius shaft or the ulna shaft were allocated to group I, while all patients with an open wedge distal radius osteotomy were allocated to group II. The main reason was that surgeons avoid planning an open wedge at the level of the diaphysis of the radius and ulna due to concerns about the healing of the osteotomies in this region. Beside the type of osteotomy the region where the osteotomy was performed (i.e. shaft or distal radius) may also had influenced the difference in the accuracy. In all but one case of Group I, where an incision of the interosseous membrane had to be performed, there was no additional soft tissue release. However, in two cases of Group II intraoperatively a considerable tension was noticed. This could also have influenced the accuracy of the achieved reduction.

Surprisingly, although not significant, the time to union was in the closing wedge/single cut osteotomy group with 4.4 months on average one month longer than in the open wedge osteotomy group. We believe there are two possible explanations for this more or less unexpected result. One the one hand the cancellous bone in the distal radius in combination with the autogenous iliac cancellous bone graft could have a positive effect on the consolidation and all open wedge osteotomies were at level of the distal radius. On the other hand in our experience in the diaphyseal region of the radius and especially of the ulna the fracture lines are regularly visible for several months or years and could have led to an overestimate of the time to union. However, we do not know the reasons therefore. Whether the necessary periosteal stripping in order to achieve a unique fitting of the guide causes a delayed union is not investigated so far.

The surgeons recognized no relevant difference in the guide fit between the groups. However, in our experience in the shaft region of the radius and ulna due to the more circular shape it can be difficult to find the exact fit. The demonstrated accuracy in performing closing wedge osteotomies at this level indicates that this was a concern in the current study.

A weakness of the study is that the CT data used in the evaluation were acquired 8–10 weeks after surgery to monitor consolidation. As a consequence the measured residual deformities might also been caused by postoperative loss of correction due to soft-tissue tensioning, weak cortical bone, delayed union, bone-plate-constructs that failed or were not rigid enough. For opening wedge osteotomies the use of structural bone graft despite the donor side morbidity [24] or a more bending resistant plate may reduce loss of correction in these cases. Another limitation is that the proposed evaluation technique can be applied only if postoperative CT data is available. Disadvantage of the actual surgical technique is that a 3D model of the implant and all screws must be available. Moreover, an uncontoured fixed-angle locking plate must be used which may fit poorly on the bone. In the future a custom-made plate [25] may be integrated in the planning application to overcome this problem.

## Conclusions

The presented in-vivo evaluation demonstrates high accuracy of the surgical technique for closing wedge and single-cut osteotomies. Whether an additional reduction guide based on K-wires may further improve accuracy in case of soft-tissue tension has to be evaluated in future studies. As the approach solely relies on the used implant, any deviation from the planned implant position or screw direction will directly influence the reduction.
